# Distance Measurement Error in Time-of-Flight Sensors Due to Shot Noise

**DOI:** 10.3390/s150304624

**Published:** 2015-02-25

**Authors:** Julio Illade-Quinteiro, Víctor M. Brea, Paula López, Diego Cabello, Gines Doménech-Asensi

**Affiliations:** 1 Centro de Investigación en Tecnoloxías da Información (CITIUS), University of Santiago de Compostela, 15782-Santiago de Compostela, Spain; E-Mails: victor.brea@usc.es (V.M.B.); p.lopez@usc.es (P.L.); diego.cabello@usc.es (D.C.); 2 Department of Electronics, Computer Technologies and Projects, Universidad Politécnica de Cartagena, 30202-Cartagena, Spain; E-Mail: gines.domenech@upct.es

**Keywords:** time-of-flight sensors, shot noise, standard CMOS technologies, distance measurement

## Abstract

Unlike other noise sources, which can be reduced or eliminated by different signal processing techniques, shot noise is an ever-present noise component in any imaging system. In this paper, we present an in-depth study of the impact of shot noise on time-of-flight sensors in terms of the error introduced in the distance estimation. The paper addresses the effect of parameters, such as the size of the photosensor, the background and signal power or the integration time, and the resulting design trade-offs. The study is demonstrated with different numerical examples, which show that, in general, the phase shift determination technique with two background measurements approach is the most suitable for pixel arrays of large resolution.

## 1. Introduction

The use of solid-state time-of-flight (ToF) sensors permits the real-time collection of 3D information of a scene without the use of high computational power or mechanical parts [1]. This type of sensor measures the time a light signal needs to travel back and forth from the target to collect the distance information. Several ToF sensors have been reported, usually classified according to the type of light signal used as modulated, if the signal is a continuous wave, or pulsed ToF, if light pulses are used.

Regardless of the measurement technique, the photosensor captures both the light signal and the background light of the scene. Given that light is a flux of discrete entities (photons), the sensing of both the light signal and background will suffer from shot noise. Shot noise is a stochastic process that can be described by Poisson statistics with a standard deviation equal to the square root of the number of photons or photogenerated charge carriers. This noise is present in the system from the beginning and, unlike other noise sources, cannot be eliminated with circuitry or signal processing techniques [2–4]. Thus, it determines the maximum achievable resolution of the ToF sensor to which the uncertainty generated by the circuitry will be added [5,6]. For the sake of comparison, the reported distance uncertainty of some ToF sensors present in the literature is shown in Table 1. As seen, the results show a great variability due to the different techniques and implementations, which complicates the evaluation of their performance. In this paper we will develop an analytic expression for the calculation of the distance error due to shot noise for each of the different existing ToF techniques, leaving aside the circuitry-related noise sources, as they depend on the particular circuit-level implementation. The study is made in terms of the size of the photosensor, the background and signal light power and the integration time. To the best of our knowledge, this is the first time that such a comprehensive study has been published for all of the pulsed ToF sensor techniques, permitting their comparison with their modulated counterparts.

The paper is organized as follows. Section 2 describes the state-of-the-art ToF implementations present in the literature. In Section 3, analytical expressions for the calculation of the distance error due to shot noise for the different modulated and pulsed ToF techniques will be developed in terms of the integration time and the reflected and background light powers, which will be considered in Sections 4 and 5, respectively. Finally, a comparison of the different ToF techniques in terms of the error in the measurement of the distance to an object for different ambient illuminations is performed in Section 6.

## 2. Related Works

The calculation of the distance by means of ToF techniques requires several measurements per period of the light signal. Due to the low light level of the received signal, these measurements need to be accumulated through several periods, reducing the attainable frame rate. Furthermore, the response of the sensing device to the light signal must be fast, in the order of tenths of nanoseconds. To fulfill all of these conditions, several designs have been proposed. In almost all of the cases, the pixel has two differentiated parts: the sensing device and one, or several, storage capacitors. This way, the flow of photocharges between the sensing device and the storage capacitors can be regulated, and different measurements can be performed.

Due to the low light levels of the signal, large sensing elements are needed, which is not compatible with the speed requirements of ToF sensors. For example, in [14], a pixel design in 0.35-μm standard CMOS technology is shown, where the sensing elements are 30 × 30 μm^2^ photogates. The storage capacitances are, in this case, four floating diffusions, and the charge flow between them and the photogate is controlled by transmission gates. It is demonstrated in this paper that, for a measurement period of 60 ns, around 50% of the photocharges are not transferred in time to the floating diffusions. Another example is [6]. Here, a 0.18-μm CMOS image sensor technology is employed for the fabrication, and a small photodiode of 9 × 9 μm^2^ is used as sensing element. With this size, the fast response needs are fulfilled, but a high number of accumulations are needed, which leads to a frame rate of only 11 frames/s.

Next, we will review the strategies that have been followed in the literature to deal with this. In some cases, special pixel designs that increase the speed transmission of the photocharges have been implemented. This allows the use of large sensing devices. Unfortunately, these pixels need to be fabricated in non-standard technologies. In other cases, standard technologies are used, which means that the sensing elements must be small to fulfill the speed requirements. In these cases, the obtained measurements are amplified using extra circuitry inside the pixel.

Regarding the designs in non-standard technologies, in [2], the ToF sensor is fabricated in CMOS/CCD technology. In this design, the sensing devices are photogates, and the acquisition of the different measurements every period is performed by controlling the transmission of the generated charges through other photogates, so they can either be integrated under an integration gate, which acts as the storage capacitance, or dumped into a dump diffusion. However, this design allows the accumulation of only one of the measurements needed for the ToF calculation. To measure the rest, the pixels must be reset and the light signal repeated. To prevent the unnecessary repetition of the light signal, in [11], the storage capacitance is duplicated. In this case, the sensing elements are also photogates, but this time, the storage capacitances are floating diffusions, and the flow of charges is controlled by transmission gates. To avoid the capture of the photocharges by the interface traps, which decreases their transmission speed, an n-buried layer is added under the photogate. This addition needs extra steps in the fabrication process. The principal problem of using photogates as sensing elements in ToF sensors is the fact that the polysilicon of the gate partially blocks the light reaching the device, hence reducing the light power of the signal more. One solution proposed for this is presented in [8], where backside-illumination is used, so that the polysilicon gate is removed from the light path.

Other designs avoid the polysilicon screening problem by using photodiodes instead of photogates as the sensing element. This is the case of [7], where the photocharges generated in a photodiode are transferred through transmission gates to two different floating diffusions that act as storage capacitors. Part of the transmission gates' length is extended over the photodiode to use their voltage to generate a horizontal electric field that accelerates the photocharge transmission to the floating diffusions, increasing the speed response of the device. Another advantage of the use of photodiodes as sensing elements is the possibility of merging in one single array ToF sensors and conventional imaging, thus obtaining a chip capable of providing color and 3D information of a scene. An example is found in [13], where an RGBZ image sensor that captures the color (Red-Green-Blue) and the depth information (Z) is presented. The pixel array alternates color acquisition pixels with ToF pixels in order to obtain, at the same time, the two types of data. Another approach is developed in [9], where the same pixels are used for color and for range acquisition. Since the needed photodiode area for the ToF sensor is bigger than that of the color image sensor, four of the image photodiodes are connected in parallel to perform the ToF operation.

If standard CMOS technologies are used, the responsivity of the sensing devices to the light signal is much lower. This means that large sensors are needed, and extra circuitry is added to increase the speed response or to amplify the sensed signal. The resultant pixels are usually bigger and with a smaller fill factor. In [12], a 0.18-μm standard CMOS technology is employed, and the sensing element used is a photodiode. Each measurement is first accumulated in the photodiode and, later, amplified and sent to the storage capacitor. As in [2], this design allows only one measurement out of the several ones needed for ToF calculation, and the pixels must be reset and the light signal repeated to perform the rest of the measurements. In [15], this problem is avoided by including two photodiodes per pixel. This way, one of them can be reset, while the other is measuring the light signal. In [10,16], extra circuitry is added in order to maintain the voltage at the photodiode node constant, making the pixel response faster. Then, the photogenerated charges are sent to the storage capacitors through transistor switches. In the first case, the technology used was a 0.6-μm (Bi)CMOS technology and, in the second one, a 90-nm standard CMOS technology.

Despite the type of pixel used, the shot noise will be present in the ToF measurement from the beginning. Some of the papers commented on above [2,6,11] present a theoretical study of this type of noise. However, the analysis is performed only for the specific technique used in those papers. In the next section, this analysis is presented for all of the existing ToF techniques, so that a comparison between them can be performed.

## 3. Shot Noise-Induced Distance Error for the Different ToF Operation Modes

**Figure 1.**
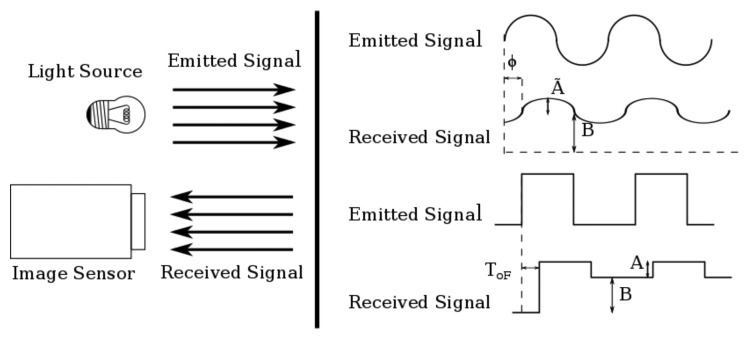
Emitted and reflected light signals in a ToF sensor.

Figure 1 shows the operation modes for modulated (top) and pulsed (bottom) ToF sensors. As seen, besides the phase or time delay, two more parameters are unknown in the incoming signal, namely its amplitude and the power of the background light of the scene. This is the reason why at least three measurements in a cycle or pulse are needed to calculate a distance. Next, we will explain the working principle of each technique and develop analytic expressions for the distance error due to shot noise in each case. In all cases, averaging by means of the accumulation of several periods of the signal for a single distance measurement to reduce shot noise is considered.

### 3.1. Modulated ToF

**Figure 2.**
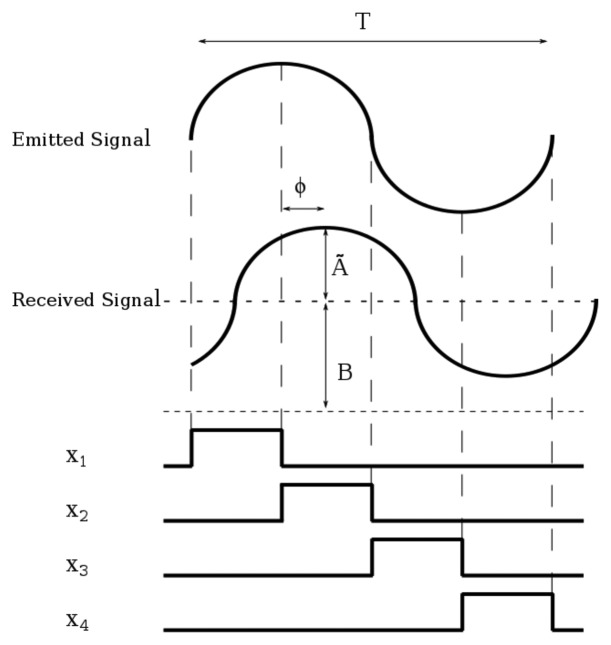
Measurements for modulated ToF.

In the modulated ToF operation the emitted signal is a continuous-wave signal modulated in time, usually a sinusoid. The phase difference between the emitted and received light signals, ϕ, depends on the distance traveled by the light, from the sensor to the object and back again. The separation between the sensor and the object can be calculated as:

(1) $$L=\frac{c}{2}\frac{\phi }{\omega }=\frac{c}{2}\frac{\phi T}{2\pi }$$

where *c* is the speed of light, *ω* the angular frequency and *T* the period of the signal. Figure 2 shows the technique to measure the phase difference. In this figure, *Ã* is the amplitude in the number of photons per second reaching the photosensor due to the signal and *B* the photons per second reaching the photosensor because of the background. Each *x_i_* measurement is the integration of these photons in the time interval *X_i_*. Four measurements are performed instead of the minimum of three to simplify the calculation. It can be demonstrated that the distance can be obtained from these measurements as [2]:

(2) $$L=\frac{c}{2}\frac{T}{2\pi }\text{atan}\frac{x_{3}-x_{1}}{x_{4}-x_{2}}$$

This equation also holds for several accumulations *N_acc_*. As the distance measurement suffers the effect of the shot noise present both in the background and in the signal, the error in the distance measurement due to this noise after the accumulation of *N_acc_* periods can be calculated by the following equation (previously published in [2]):

(3) $$\Delta L_{\text{mod}}=\frac{c}{2}\frac{T\sqrt{B}}{2\sqrt{8}\tilde{A}\sqrt{N_{\text{acc}}}}$$

### 3.2. Pulsed ToF

Pulsed ToF employs square wave signals. The time delay between the emitted and received light pulses, *T_oF_*, depends on the distance traveled by the light, which can be calculated as:

(4) $$L=\frac{c}{2}T_{oF}$$

Two measurement techniques exist for the determination of the distance using pulsed ToF, phase shift determination (PSD) and multiple double short time integration (MDSI). As in the case of modulated ToF, *A* and *B* are the numbers of photons per second hitting the photosensor because of the signal and the background, respectively.

#### 3.2.1. Phase Shift Determination

**Figure 3.**
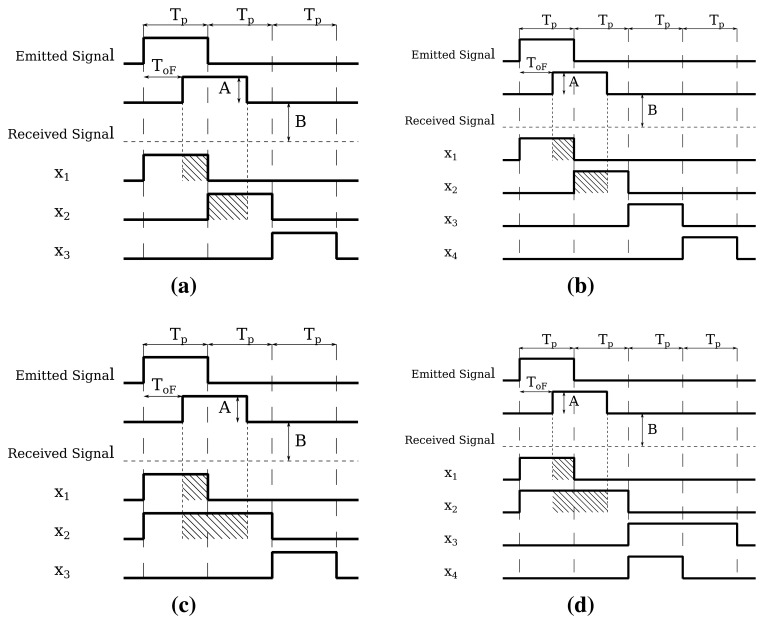
Measurements for pulsed ToF: (**a**) phase shift determination with one background measurement (PSD-1B); (**b**) PSD-2B; (**c**) multiple double short time integration (MDSI)-1B; (**d**) MDSI-2B.

In the PSD case, all of the measurements have a duration equal to that of the emitted pulse, *T_p_*. From Figure 3, it can be seen that the PSD technique can be realized in two different ways, with either three (Figure 3a) or four measurements (Figure 3b). In both cases, the first measurement interval, *X*_1_, is synchronized with the emitted pulse, while the second, *X*_2_, comes right after it. The third and fourth measurements are carried out without the light signal in order to sense the background light. We will refer to the technique depicted in Figure 3a as PSD with one background measurement (PSD-1B) and to the one in Figure 3b as PSD with two background measurements (PSD-2B). 1B techniques require three storage elements, one for each *x_i_*, while for 2B, only two are needed: one for *x*_1_ − *x*_3_ and the other one for *x*_2_ − *x*_4_. As these values are usually stored in capacitors, the capacitances of 2B procedures are much smaller, since the background is eliminated before the storage. For PSD-1B, it is easy to show that the number of photons in each measurement interval is,

(5a) $$x_{1}=BT_{p}+A\left(T_{p}-T_{oF}\right)$$

(5b) $$x_{2}=BT_{p}+AT_{oF}$$

(5c) $$x_{3}=BT_{p}$$

From Equation (5), the *T_oF_* can be calculated, and the distance will be:

(6) $$L_{\text{PSD}-1B}=\frac{c}{2}T_{p}\frac{x_{2}-x_{3}}{\left(x_{1}-x_{3}\right)+\left(x_{2}-x_{3}\right)}$$

As in the case of modulated ToF, this equation does not change with the accumulation of *N_acc_* pulses. Applying error propagation,

(7) $$\Delta L=\sqrt{\sum_{i}{\left(\frac{\partial L}{\partial x_{i}}\right)}^{2}{\left(\delta x_{i}\right)}^{2}}$$

and knowing that the *x_i_* signals follow a Poisson distribution, (*δx_i_*)^2^ = *x_i_*, the uncertainty for the distance due to the shot noise can be calculated as:

(8) $$\Delta L_{\text{PSD}-1B}=\frac{c}{2}\frac{\sqrt{2BT_{p}^{2}-\left(6B-A\right)T_{oF}\left(T_{p}-T_{oF}\right)}}{A\sqrt{N_{\text{acc}}}\sqrt{T_{p}}}$$

A more detailed calculation of Equation (8) is included in Appendix A. In [6,11], a similar analysis of the shot noise is presented, but in both cases, the background effect is neglected, yielding less accurate expressions.

For the PSD-2B case, Equations (5a)–(5c) are modified as:

(9a) $$x_{1}=BT_{p}+A\left(T_{p}-T_{oF}\right)$$

(9b) $$x_{2}=BT_{p}+AT_{oF}$$

(9c) $$x_{3}=BT_{p}$$

(9d) $$x_{4}=BT_{p}$$

and the distance calculation changes to,

(10) $$L_{\text{PSD}-2B}=\frac{c}{2}T_{p}\frac{x_{2}-x_{4}}{\left(x_{1}-x_{3}\right)+(x_{2}-x_{4})}$$

Using Equation (7), the uncertainty in the distance calculation caused by the shot noise in the PSD-2B case is given,

(11) $$\Delta L_{\text{PSD}-2B}=\frac{c}{2}\frac{\sqrt{2BT_{p}^{2}-\left(4B-A\right)T_{oF}\left(T_{p}-T_{oF}\right)}}{A\sqrt{N_{\text{acc}}}\sqrt{T_{p}}}$$

#### 3.2.2. Multiple Double Short Time Integration

In the MDSI technique, the first measurement is also synchronized with the emitted pulse and has the same duration, while for the second, its duration is doubled in order to ensure that the entire received pulse is measured during *X*_2_. Like in PSD, the third and fourth measurements are performed without light signal to sense the background. These measurements are shown in Figure 3c for the MDSI-1B and in Figure 3d for the MDSI-2B.

For MDSI-1B, the number of photons at the photosensor, *x_i_*, in each measurement interval *X_i_* can be extracted from Figure 3c as:

(12a) $$x_{1}=BT_{p}+A\left(T_{p}-T_{oF}\right)$$

(12b) $$x_{2}=2BT_{p}+AT_{p}$$

(12c) $$x_{3}=BT_{p}$$

Using Equations (12a)–(12c) and (4), the distance can be calculated as:

(13) $$L_{\text{MDSI}-1B}=\frac{c}{2}T_{p}\frac{x_{2}-x_{1}-x_{3}}{x_{2}-2x_{3}}$$

Again, this equation is valid for any number of pulses *N_acc_*. The distance uncertainty because of the shot noise can be calculated by error propagation from Equation (7) as:

(14) $$\Delta L_{\text{MDSI}-1B}=\frac{c}{2}\frac{\sqrt{\left(4B+2A\right)T_{p}^{2}+\left(6B+A\right)T_{oF}^{2}-\left(8B+3A\right)T_{p}T_{oF}}}{A\sqrt{N_{\text{acc}}}\sqrt{T_{p}}}$$

For the MDSI-2B technique, Equations (12a)–(12c) are modified to take into account the extra background measurement:

(15a) $$x_{1}=BT_{p}+A\left(T_{p}-T_{oF}\right)$$

(15b) $$x_{2}=2BT_{p}+AT_{p}$$

(15c) $$x_{3}=BT_{p}$$

(15d) $$x_{4}=2BT_{p}$$

The distance and its uncertainty due to shot noise become:

(16) $$L_{\text{MDSI}-2B}=\frac{c}{2}T_{p}\frac{\left(x_{2}-x_{4}\right)-\left(x_{1}-x_{3}\right)}{x_{2}-x_{4}}$$

(17) $$\Delta L_{\text{MDSI}-2B}=\frac{c}{2}\frac{\sqrt{\left(6B+2A\right)T_{p}^{2}+\left(4B+A\right)T_{oF}^{2}-\left(8B+3A\right)T_{p}T_{oF}}}{A\sqrt{N_{\text{acc}}}\sqrt{T_{p}}}$$

From Equations (3), (8), (11), (14) and (17), it can be inferred that the error in the distance caused by the shot noise is not only affected by the power of the light signal and background, but also by the number of accumulations, the duration of the light pulse and even the value of *T_oF_*. The error will increase with bigger *B* and *T_p_* values, and it will decrease with bigger A and *N_acc_*. The effect of the latter will be studied in Section 4, whereas light power considerations for the calculation of *A* and *B* will be made in Section 5. On the other hand, the comparison of Equations (8), (11) (14) and (17) shows that 1B techniques present lower shot noise than the 2B ones. This will be addressed with numerical examples in Section 6.

## 4. Signal Accumulation

**Figure 4.**
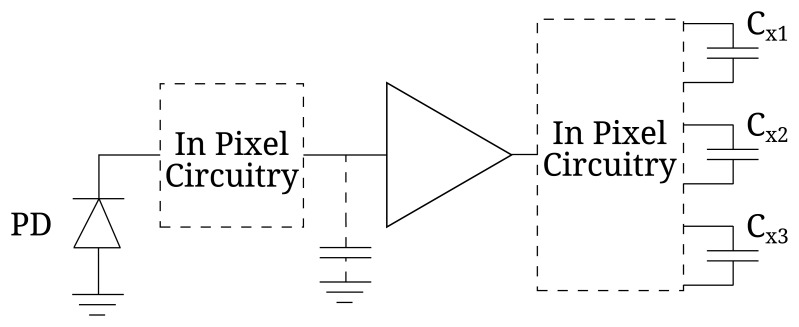
Schematic of a voltage mode pixel for 1B techniques.

**Figure 5.**
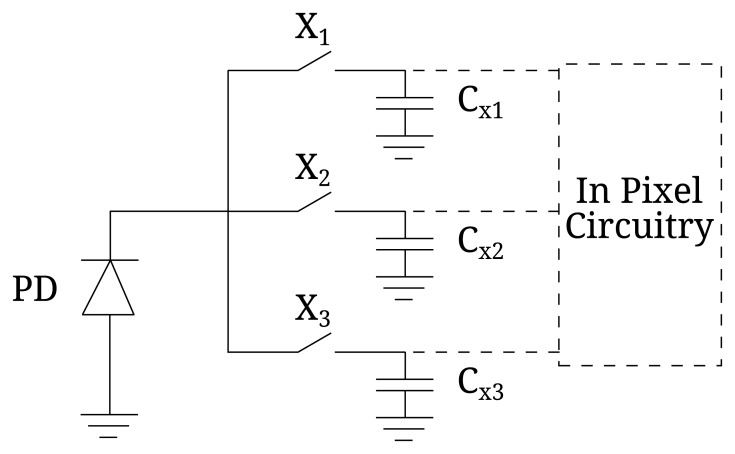
Schematic of a current mode pixel for 1B techniques.

As explained, regardless of the ToF technique, signal averaging by means of several accumulations is needed in order to reduce the shot noise of a single distance measurement and, consequently, Δ*L*. In this section, we will calculate the value of *N_acc_* for each ToF technique in a given integration time, *T_int_*, defined as the period of time during which the *x_i_* samples are accumulated. To do so, it is necessary to take into account that the ToF pixel can be implemented in either voltage or current mode, shown respectively in Figures 4 and 5 for 1B techniques, without loss of generality. In voltage mode, the photocharges of each *x_i_* measurement are accumulated in an intermediate capacitor (which can be the photosensor itself) transforming the charge signal into a voltage one, [14,15]. This mode of operation has the disadvantage of requiring the resetting of the intermediate capacitor before each *x_i_* measurement. In the current mode, the photosensor works at a constant voltage, and the generated photocharges are transferred directly to the storage capacitors, making the resetting between measurements unnecessary [6,16].

**Figure 6.**
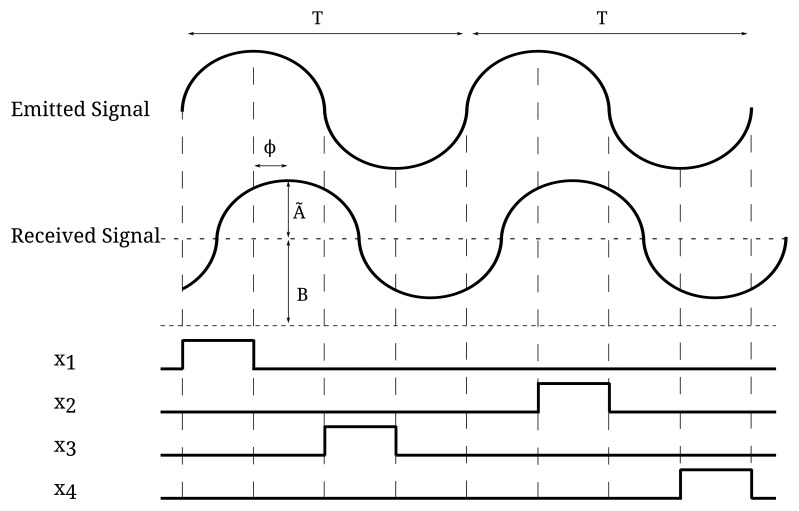
Sketch of the measurements needed in voltage mode-modulated ToF.

To calculate *N_acc_*, we first define *T* as the period of the emitted signal in modulated ToF or, alternatively, the time between two consecutive light pulses in pulsed ToF techniques; and *N_LP_* as the number of light pulses needed to perform the measurement of all of the *x_i_* parameters. The value of *N_LP_* depends on the particular ToF technique and whether it is implemented in current or voltage mode. Figure 2 shows the measurement intervals in current mode-modulated ToF operation. As seen, all of the *x_i_* measurements needed for a cycle can be measured in a period *T*, and thus, *N_LP_* = 1. However, in voltage mode-modulated ToF, represented in Figure 6, a reset operation is needed between consecutive *x_i_*, and hence, *N_LP_* = 2. The same holds for PSD. In the MDSI case, *N_LP_* = 2 in both voltage and current mode, because *x*_1_ and *x*_2_ overlap and cannot be measured in the same pulse; see Figure 3. The number of accumulations per distance measurement depends on these three parameters in the following manner,

(18) $$N_{\text{acc}}=\frac{T_{\text{int}}}{N_{LP}T}$$

Table 2 summarizes the number of accumulations for every technique. As seen, increasing the integration time can lead to a reduced shot noise, as it increases the number of signal accumulations for a given ToF technique and frequency of the emitted signal.

## 5. Light Power Considerations

In ToF operation, the light signal emitted by the source will reach the sensor after being reflected by the target. In order to calculate Δ*L* for the different ToF techniques, it is necessary to determine the amplitude in the number of photons reaching the photosensor due to the reflected signal, *A* and *Ã* for pulsed and modulated ToF, respectively, and the number of photons due to the background light, *B*, in terms of the emitted light power source; see Figure 1. The average light power that reaches the target is determined by eye safety regulations [17], the maximum average light power per surface area that can reach the eye without safety glasses limited to around 1 mW/cm^2^. After hitting the target, the light power density reflected at the target can be calculated as,

(19) $$p_{d}\left(L\right)=\frac{\rho P_{\text{light}}}{{\left(2L\text{tan}\left(\theta /2\right)\right)}^{2}}$$

where *ρ* is the target reflectivity, *P_light_* the light source power, *L* the distance between the source and the target and θ the emitter beam divergence. After being reflected by the target, this light signal hits the sensor. The light power density at the pixel, *p_pix_*(*L*), can be calculated as [7],

(20) $$p_{\text{pix}}\left(L\right)=\frac{\tau_{\text{opt}}p_{d}\left(L\right)}{4F{\#}^{2}}$$

where *τ_opt_* is the optics transmission efficiency and *F#* the F-number. After obtaining the light power density at the sensor site from a specific distance, it can be extended to any distance as,

(21) $$p_{\text{pix}}\left(L_{2}\right)=p_{\text{pix}}\left(L_{1}\right){\left(\frac{L_{1}}{L_{2}}\right)}^{2}$$

It is important to distinguish between instant and average light power reaching the pixel. In modulated ToF, since the light signal is a sinusoidal wave, the average light power density reaching the pixel, *p̄_pix_*, equals its maximum instant light power density, that is,

(22) $${{\bar{p}}_{\text{pix}}|}_{\text{mod}}=p_{\text{pix},\max}$$

However, in the pulsed case, the instant light power density at the pixel has a value of *p_pulse_* during the duration of the pulse and zero for the rest of the time. As a result, the instant light power density on the pulsed ToF can be determined as,

(23) $$p_{\text{pulse}}={\bar{p}}_{\text{pix}}\frac{T}{T_{p}}$$

With Equations (20)–(23), the values of *Ã* and A can be calculated as:

(24) $$\tilde{A}=\frac{p_{\text{pix},\text{max}}A_{PS}\lambda }{hc}$$

(25) $$A=\frac{p_{\text{pulse}}A_{PS}\lambda }{hc}$$

where *A_PS_* is the area of the photosensor, λ the wavelength of the incident light and *h* Planck's constant. As can be seen, *Ã* is limited by the maximum allowed average power, which is, in turn, related to the maximum light source power determined by eye safety regulations, while *A* can be increased by increasing *T*.

Finally, *B* can be calculated from the instant light power density of the background, which is assumed to be constant and equal to the average background power density, *p̄_B_*. This value can be calculated from typical values of luminance and conversion factors; see, for example, [18]. Thus,

(26) $$B=\frac{{\bar{p}}_{B}A_{PS}\lambda }{hc}$$

## 6. Comparison of the Different ToF Techniques

### 6.1. Numerical Examples

In this section, numerical examples comparing the different ToF solutions are presented. These examples are intended to be as general as possible, but some values must be set. In particular, the wavelength of the light signal is set to λ = 850 nm. Furthermore, in all cases, we used *T* = 50 ns and *T_p_* = 50 ns in modulated and pulsed ToF, respectively, which means that the maximum distance measurable by the sensor is 7.5 m (Equations (1) and (4)). The integration time was set to *T_int_* = 20 ms, during which many accumulations of *x_i_* are stored in the pixel. This leaves more than 10 ms for A/D conversion and read-out to comply with the time constraint of the video rate, 33 frames per second. Finally, the area of the photosensor is set to 15 × 15 μm^2^. These assumptions affect the final values, but not the relationship between the different ToF techniques.

**Figure 7.**
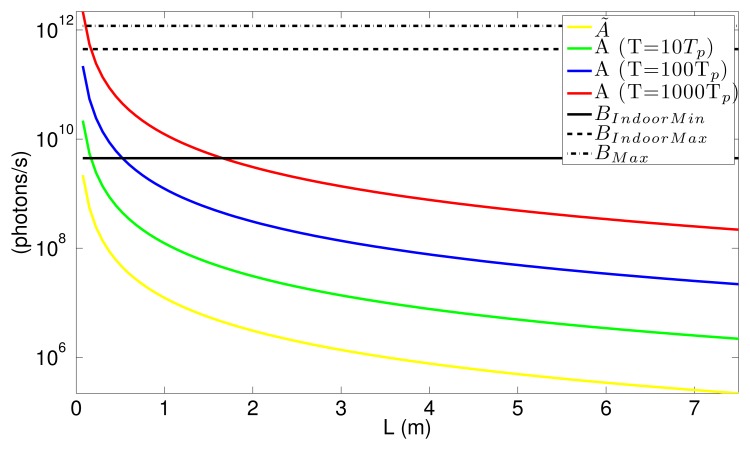
*Ã* and *A* values as a function of the distance between the target and the sensor. A values are shown for three different *T*.

Three different ambient light illuminations were studied: a poorly-illuminated indoor scenario, with a background light power density of *p̄BIndoorMin* = 6.25 × 10^−4^ W/cm^2^; a well-illuminated indoor scenario with *p̄BIndoorMax* = 6.25 × 10^−2^ W/cm^2^; and outdoor illumination in midsummer with *p̄BMax* = 0.167 W/cm^2^. This background is comprised of light of different wavelengths, but for the calculation of B using Equation (26), λ = 630 nm was used, since around this wavelength, the silicon has higher sensitivity. The obtained values are: *B_IndoorMin_* = 4.46 × 10^9^, *B_IndoorMax_* = 4.46 × 10^11^ and *B_Max_* = 1.19 × 10^12^ photons per second. Assuming that the eye safety regulations are satisfied for every distance to the sensor greater than 10 cm, the light power per unit area impinging the sensor at this distance is around *p̄_pix_*(10 cm)= 130 μW/cm^2^. In this calculation, we set *ρ* = 0.5, θ = 40°, *τ_opt_* = 1 and *F#* = 1.4. From this, and using Equations (21) and (24), the maximum value of *A* as a function of the distance between the sensor and the target can be calculated; Figure 7. As can be seen, in modulated ToF techniques, *Ã* is severely reduced for longer distances, which, in turn, results in a higher shot noise. On the other hand, in pulsed ToF, the value of *A* depends on *T* through *p_pulse_*. Longer *T* values result in higher *A*, due to the fact that by increasing *T*, it is possible to increase the light power of the pulse without increasing the average light power, as seen in Equation (23). It should also be noted that in modulated ToF, the number of photons reaching the sensor because of the background is bigger than those from the signal in all of the background situations. In pulsed ToF, even with large *T* values, the situation is only reversed for distances lower than 2 m.

**Figure 8.**
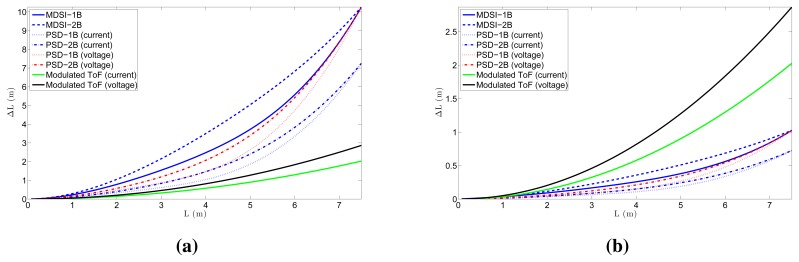
Distance error due to shot noise for the different ToF techniques. In pulsed ToF, *T* was set to: (**a**) T = 0.5 μs (10*T_p_*); (**b**) T = 50 μs (1000*T_p_*).

With these values, the distance error due to shot noise can be calculated. Figure 8a,b shows the distance error due to the shot noise for all of the different ToF techniques for *B_IndoorMin_*. Values of *T* =0.5 μs (10*T_p_*) and *T* =50 μs (1000*T_p_*) were used for the pulsed-ToF techniques, respectively, whereas *T* = 50 ns was used for modulated ToF. By comparing both figures, the fact that the shot noise in pulsed ToF is reduced with larger *T* is apparent, despite the fact that the number of accumulations is reduced. The reason for this is that, as seen in Equation (23), increasing *T* results in larger instant light power for a given value of *p̄_pix_*, which is usually limited by eye safety constraints. It can also be seen that, because of the reduction of *Ã* and *A* for greater distances, the shot noise and, thus, the accuracy worsen with the distance. In addition, a comparison between ToF techniques can be performed. First of all, for the same ToF technique, the current mode always presents less error due to shot noise than the voltage one, except in the MDSI case, where they are the same. The reason for this can be inferred from Table 2. The number of accumulations in both modulated and PSD ToF for a given integration time in the current mode is twice the number as in the voltage one, because, in the latter, two light pulses per measurement are needed. The MDSI technique needs two light pulses per measurement in both modes, so that the shot noise does not change. In addition, by comparing PSD and MDSI, it can be seen that the first one is better, as it requires less light pulses per measurement. Finally, for the same technique, 1B measurements present lower shot noise than 2B measurements.

**Figure 9.**
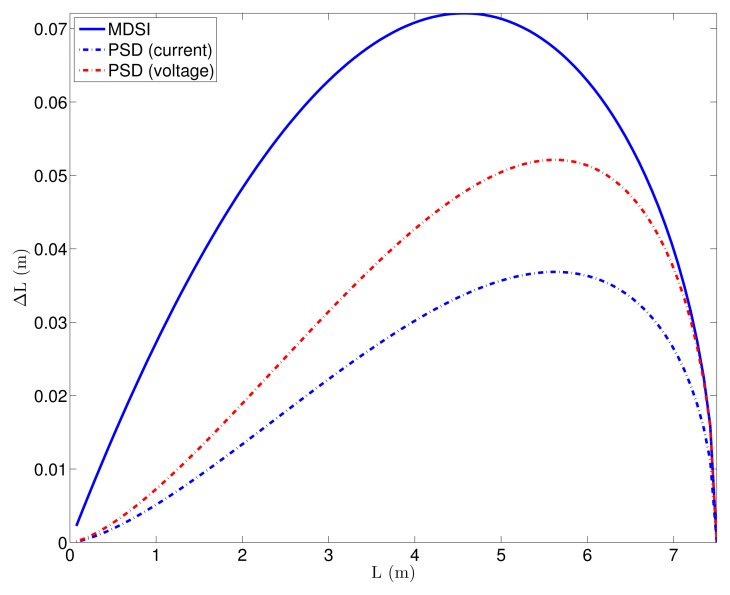
Distance error due to shot noise for the different ToF techniques without background noise. In the pulsed ToF techniques, *T* was set to 50 μs (1000*T_p_*).

The errors in the distance measurement in the situations depicted in Figure 8a,b are too high for most applications. There exist four ways of reducing these values. The first one is to increase the light power of the pulse; however, this will violate eye safety regulations. The second option is to use larger photosensors, as increasing the photosensor area by a factor of four reduces the shot noise uncertainty by two, but larger photosensors have slower responses. The third option is to increase the integration time of the sensor with a fixed *T*, which increases *N_acc_* and, hence, reduces Δ*L* by a factor $1/\sqrt{N_{\text{acc}}}$. Alternatively, for pulsed ToF, increasing the integration time permits one to increase *T* and, thus, A, which reduces the distance uncertainty as $\Delta L\propto \sqrt{A}/A$. Finally, the last option is to minimize the background light reaching the pixel, which, in practice, is usually accomplished by placing optical filters in front of the sensor that restrict the incident light to the wavelength of the light source. The same situation as in Figure 8b, but without background noise (*B* = 0), is shown in Figure 9. This represents the minimum achievable error of the ToF sensor. In the ideal situation of no background light, the modulated ToF technique does not have shot noise error, and there is no difference between the 1B and 2B measurement techniques.

### 6.2. Adaptive Number of Accumulations

**Figure 10.**
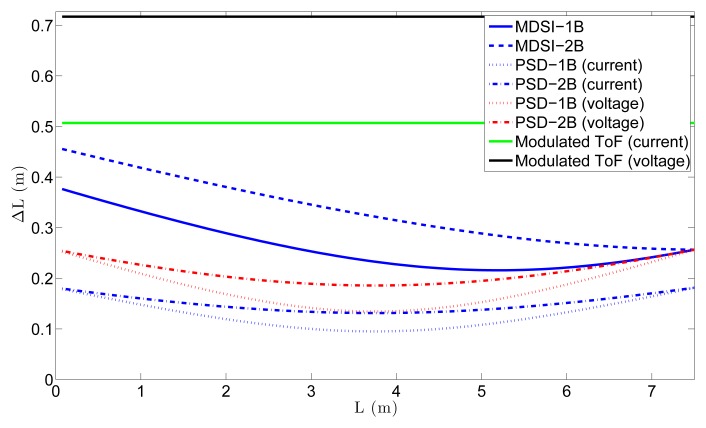
Distance error due to shot noise for the different ToF techniques with an adaptive number of accumulations and *B_Indoor Min_*. In the pulsed ToF techniques, *T* was set to 50 μs (1000*T_p_*).

The decrease of the power of the light signal reaching the sensor with the distance not only increases the shot noise uncertainty for long distances, but also generates big differences in the signal-to-noise ratio (SNR) of the pixels and, therefore, in the fixed pattern noise (FPN) [19]. A solution proposed in [15] is to use an adaptive number of accumulations, where *T_int_* of each pixel is not fixed. Instead, a pixel continues to accumulate measurements until a certain voltage in the storage capacitors is reached. This is equivalent to the situation where the light signal power reaching the pixel, that is either *Ã* or *A*, is constant for every distance. Figure 10 shows the shot noise uncertainty as a function of the distance when *A* is maintained constant and equal to A at 3.75 m. The figure shows that with the adaptive number of accumulations, the error introduced by the shot noise is constant for the modulated ToF techniques and shows smaller differences in the pulsed ToF ones.

### 6.3. Selection Criteria

The results above seem to suggest that the 1B methods should be preferred over the 2B ones, as they suffer less shot noise. However, the 2B solutions have the advantage of a reduction in the size of the storage capacitors, which is determined by the maximum number of electrons to be stored in a single measurement, *N_emax_*. This value depends on the particular ToF technique and, for example, for PSD-1B Equation (5a), shows that, for *x*_1_, the maximum number of generated electrons occurs when *T_oF_* = 0, *N_emax_* = η(*BT_p_* + *AT_p_*), where η is the quantum efficiency of the photosensor. Similar considerations can be made for the rest of the techniques. Given a maximum voltage swing allowed in the capacitor, *V_cmax_*, and the number of accumulations, the minimum storage capacitance can be calculated as:

(27) $$C_{x_{i},\text{min}}=\frac{qN_{\text{acc}}}{V_{c\max}}N_{e\max}$$

The actual number and size of the capacitors depend on the measuring technique. Both modulated and 2B-pulsed techniques need only two capacitors, whereas 1B-pulsed techniques need three. Besides, since 1B techniques accumulate *A* and *B*, such capacitors are bigger, too. Therefore, the 1B approach will lead to smaller shot noise, but at the cost of bigger pixels with a smaller fill factor.

In Table 3, examples of the capacitor values needed for each technique are listed. For the calculation of these values, the quantum efficiency of the photosensor has to be set. In these examples, an n-well over p substrate photodiode in 0.18-μm standard CMOS technology was supposed, and the η values were obtained from [20]. Furthermore, *V_cmax_* = *V_dd_* =1.8 V. In addition, the current mode was considered, and a background of *B_Indoor Max_* was used. As can be seen, capacitors in 1B solutions are nearly two-times bigger than their 2B counterparts. Moreover, a third capacitor is needed for the background storage. If the voltage mode is considered instead, the capacitances needed are half the ones in Table 3, except for the MDSI techniques, which need the same storage capacitors in both modes. These capacitance values are high, because they are designed to avoid saturation, even if the signal is reflected from an object very near to the sensor (a high number of photons reaching the pixel), and the pixel operates in the course of the entire *T_int_*. If the range of measurable distances starts at longer distances, that is an interval close to the sensor is ignored, these capacitances can be decreased. Furthermore, if an adaptive number of accumulations is used, the capacitances can be reduced, too, since the pixel operation is always interrupted before the value stored in the capacitor reaches its maximum possible value.

## 7. Conclusions

A theoretical study of the error in the distance measurement for all of the different ToF sensors due to shot noise has been performed. The study shows that, under normal illumination conditions, the pulsed ToF techniques perform better than the modulated ones. Furthermore, within the pulsed ToF techniques, PSD presents lower noise than MDSI. Regarding the measurement mode, the current mode has less shot noise than the voltage mode. Finally, despite the fact that the noise is reduced when 1B techniques are employed, the increase in the storage capacitances makes, in general, the 2B approach more suitable for pixel arrays of large resolution, in the order of tensof thousands of pixels.

## Figures and Tables

**Table 1.** Comparison between different experimental results presented in the literature for ToF sensors.

	**[7]**	**[8]**	**[9]**	**[10]**	**[11]**	**[12]**	**[13]**
Photosensor (μm^2^)	4.9 × 4.9	14 × 14	7 × 7	101 × 101	6.5 × 6.5	17 × 17	2.25 × 9
Integration time (ms)	200	66	50	50	-	19	10
Frequency/pulse width	20 MHz	20 MHz	50 ns	50 ns	100 ns	50 ns	50 ns
Light power	80 mW	6.6 W/m^2^ @1 m	1100 mW	900 mW	0.29 W/m^2^ @ 1 m	89 mW	450 mW
Distance error	16 cm @ 6 m	5 cm @ 4 m	4.3 cm @ 3 m	1 cm @ 1 m	3 cm @ 1 m	40 cm @ 3 m	3.8 cm @ 4.5 m

**Table 2.** Number of accumulations for different ToF techniques.

	**Number of Accumulations**, *N_acc_*	

**Measurement Technique**	**Current Mode**	**Voltage Mode**
Modulated ToF	$\frac{T_{\text{int}}}{T}$	$\frac{T_{\text{int}}}{2T}$
PSD	$\frac{T_{\text{int}}}{T}$	$\frac{T_{\text{int}}}{2T}$
MDSI	$\frac{T_{\text{int}}}{2T}$	$\frac{T_{\text{int}}}{2T}$

**Table 3.** Minimum capacitance for all of the ToF measurement techniques.

**Technique**	**1st Signal C (fF)**	**2nd Signal C (fF)**	**Background C (fF)**
Modulated ToF	156.0	156.0	-
PSD-1B	722.1	722.1	346.1
PSD-2B	376.0	376.0	-
MDSI-1B	361.1	534.1	173.1
MDSI-2B	188.0	188.0	-

## A. Appendix Calculation of the Shot Noise-Related Error in PSD-1B

This Appendix shows how to analytically derive the expressions of the distance uncertainty of Section 3 through error propagation due to the Poisson distribution of the incoming light. A thoroughderivation for the PSD-1B technique is addressed. The procedure is similar for PSD-2B, MDSI-1B and MDSI-2B. The measurements necessary for the calculation of the ToF in the PSD-1B technique are explained in Figure 3. Equation (5) describes the number of photons reaching the pixel during each of the *X_i_* measurement intervals. This equation is rewritten here to include the fact that the measurement is repeated *N_acc_* times:

(A1a) $$x_{1}=N_{\text{acc}}\left[BT_{p}+A\left(T_{p}-T_{oF}\right)\right]$$

(A1b) $$x_{2}=N_{\text{acc}}\left[BT_{p}+AT_{oF}\right]$$

(A1c) $$x_{3}=N_{\text{acc}}\left[BT_{p}\right]$$

Subtracting Equation (A1c) from Equations (A1a) and (A1b), the equations shown below are obtained:

(A2a) $$x_{1}-x_{3}=N_{\text{acc}}A\left(T_{p}-T_{oF}\right)$$

(A2b) $$x_{1}-x_{3}=N_{\text{acc}}AT_{oF}$$

(A3) $$x_{2}+x_{3}-2x_{3}=N_{\text{acc}}AT_{p}$$

From these equations, the *T_oF_* is obtained as:

(A4) $$T_{oF}=T_{p}\frac{x_{2}-x_{3}}{x_{1}+x_{2}-2x_{3}}$$

The shot noise-related error present in the ToF can be calculated by error propagation:

(A5) $$\delta T_{oF}^{2}={\left(\frac{\partial T_{oF}}{\partial x_{1}}\right)}^{2}\delta x_{1}^{2}+{\left(\frac{\partial T_{oF}}{\partial x_{2}}\right)}^{2}\delta x_{2}^{2}+{\left(\frac{\partial T_{oF}}{\partial x_{3}}\right)}^{2}\delta x_{3}^{2}$$

From Equation (A1), the partial derivative of *T_oF_* with respect to the different *x_i_* $\frac{\partial T_{oF}}{\partial x_{i}}$, is,

(A6a) $$\frac{\partial T_{oF}}{\partial x_{1}}=T_{p}\frac{-\left(x_{2}-x_{3}\right)}{x_{1}+x_{2}-2x_{3}}=T_{p}\frac{-N_{\text{acc}}AT_{oF}}{{\left(N_{\text{acc}}AT_{p}\right)}^{2}}$$

(A6b) $$\frac{\partial T_{oF}}{\partial x_{2}}=T_{p}\frac{x_{1}-x_{3}}{x_{1}+x_{2}-2x_{3}}=T_{p}\frac{N_{\text{acc}}A\left(T_{p}-2T_{oF}\right)}{{\left(N_{\text{acc}}AT_{p}\right)}^{2}}$$

(A6c) $$\frac{\partial T_{oF}}{\partial x_{3}}=T_{p}\frac{-\left(x_{1}-x_{2}\right)}{x_{1}+x_{2}-2x_{3}}=T_{p}\frac{-N_{\text{acc}}A\left(T_{p}-2T_{oF}\right)}{{\left(N_{\text{acc}}AT_{p}\right)}^{2}}$$

On the other hand, because the flux of photons reaching the device can be described by Poisson statistics, the variance in the *x_i_* values are:

(A7a) $$\delta x_{1}=x_{1}=N_{\text{acc}}\left[BT_{p}+A\left(T_{p}-T_{oF}\right)\right]$$

(A7b) $$\delta x_{2}=x_{2}=N_{\text{acc}}\left[BT_{p}+AT_{oF}\right]$$

(A7c) $$\delta x_{3}=x_{3}=N_{\text{acc}}\left[BT_{p}\right]$$

Replacing Equations (A6) and (A7) in Equation (A5) and operating, the next equation is obtained:

(A8) $$\delta T_{oF}=\frac{\sqrt{2BT_{p}^{2}-\left(6B-A\right)T_{oF}\left(T_{p}-T_{oF}\right)}}{A\sqrt{N_{\text{acc}}}\sqrt{T_{p}}}$$

Replacing Equation (A8) in Equation (4), the error in the distance measurement because of the shot noise is obtained:

(A9) $$\Delta L_{\text{PSD}-1B}=\frac{c}{2}\frac{\sqrt{2BT_{p}^{2}-\left(6B-A\right)T_{oF}\left(T_{p}-T_{oF}\right)}}{A\sqrt{N_{\text{acc}}}\sqrt{T_{p}}}$$

The calculation for the rest of the ToF techniques is analogous to the one explained in this Appendix.
